# Medical Humanities: time to join the mainstream of medical education

**DOI:** 10.15694/mep.2018.0000182.1

**Published:** 2018-09-03

**Authors:** Muiris Houston

**Affiliations:** 1 TCD

**Keywords:** Medical Humanities, narrative medicine, patient stories, patient centred care, curriculum development, professionalism

## Abstract

This article was migrated. The article was marked as recommended.

Medical Humanities has grown in stature and scholarship in recent years. Much of the credit for this must go to academic humanists. Medical academics, with some notable exceptions, have not taken ownership of the discipline. The time has come for this to change. The medical humanities have so much to offer students in this era of patient centred healthcare that this imbalance must be addressed. It’s time for medical humanities to move from the periphery of medical education to a central role in the undergraduate curriculum.

## Introduction

There is most definitely a need to bring the humanities to a more central position within medical education. Why do so many medical humanities modules languish on the periphery in elective and special study modules? Is there an over reliance on humanities departments and not enough involvement from medical schools?

I would like to use this paper to argue, through the prism of narrative medicine, how the medical humanities would benefit from a rebalancing of its influences and drivers.

## Discussion

As the authors of the editorial in this theme issue have pointed out, (quoting Frenk et al), “professional education has not kept pace with challenges [in healthcare delivery] largely because of fragmented, out-dated, and static curricula that produce ill-equipped graduates” ( Frenk et al., 2010) ( McFarland et al 2018).

In its 2010 report on medical education in the US, the Carnegie Foundation found the system to be “inflexible, overly long and not learner- centred.” It noted a lack of holistic learning about patients’ experiences, and perhaps most disturbingly that “the pace and commercial nature of healthcare often impede the inculcation of fundamental values of the profession.” In short, a technocratic, reductionist system had taken hold. ( Cooke et al 2010).

However all is not lost. The Core Curriculum for Sociology in UK Undergraduate Medical Education ( BeSST, 2018), produced by the Behavioural & Social Sciences Teaching in Medicine (BeSST) Sociology Steering Group brings a renewed emphasis on an authentic curriculum based on real medical practice.

Medical humanities have a crucial role in “humanizing” the future practitioner. As underlined in this theme edition’s editorial, the human condition cannot be fully understood by scientists. Iona Heath, a long- standing advocate for narrative medicine in the UK states: “Most clinicians are not scientists; they have a different responsibility – to attempt to relieve distress and suffering ..” ( Heath, 2016)

In their recently published paper in this journal, “ Integrating humanities curricula in medical education: a literature review”, Taylor, Lehmann and Chisholm pinpointed a weakness in how medical humanities are currently taught:

“Most of the curricula were described as running separately to biomedical teaching on areas such as pathology, biochemistry or physiology. They were often taught by arts educators, without clinician involvement. This could limit the potential for students to understand how humanities can contribute to all areas of medicine as opposed to simply communication or writing skills.”

The authors posit that adopting an integrated collaborative approach to teaching, with arts educators working alongside clinicians could help to bridge the gap between science education and humanities education. ( Taylor et al, 2018)

For that to actually happen, however, would mean a much greater involvement in, and commitment to, the medical humanities by mainstream medical educators. Without prioritisation of resources and a restructuring of medical school curricula, there is a not insubstantial risk of medical humanities continuing to be a “Cinderella” discipline, languishing on the periphery of medical education.

In criticizing the dearth of clinicians involved in teaching medical humanities, the contribution of academic humanists must be lauded. Indeed without their extended scholarship, medical humanities could not have developed as it has. It is a sign of maturity on the part of these academics that they have reached a point where reflective criticism has emerged. Perhaps the most comprehensive avenue of criticism suggested for narrative medicine comes from Angela Woods in the Journal of Medical Ethics: Medical Humanities. She describes some seven dangers in the dominant medical humanities approach to narrative including the core issue of whether all human beings are “naturally narrative”. It leads to the question: is narrative always healthy and good in the case of illness? ( Woods 2011)

Among her specific concerns are the extent to which we can we trust that people’s stories of illness faithfully describe “what it is really like”; is there a danger in overinflating what counts as narrative; and practitioners working with narrative in medicine often overlook the cultural and historical dimensions of the narrative form.

Responding to Woods challenge,“to reignite critical debates” around the limits of narrative, McKechnie looks to identify ways to re-place narrative at the centre of medical practice by “reassessing the role of the narratee in the narrative process.” Defining narrative in terms of the listener, McKechnie avers that however loosely based on fact, the narratee formulates a story to make sense of what they see, hear and experience. However, McKechnie also cautions that the narrative that takes shape in the health professional’s mind may not be an accurate reflection of the patient’s account of what has happened to them. ([ McKechnie 2014) Acknowledging the complexity and intricacy of narrative, she concludes:

“the boundaries of narrativity, then, should be expanded to include those forms of expression that Woods refers to as non-narrative because even (and sometimes especially) non-verbal expression requires language and narrative ordering in the construction of expression and in the process of meaning making.”

Wald advocates the introduction of narrative medicine initiatives in the Pre-clinical year ‘getting their [students] feet wet,’-with the aim of cultivating more sophisticated reflection skills in the clinical years and

beyond. (Wald 2011). This was one of the guiding principles when developing teaching modules for medical students in Trinity College Dublin and the National University of Ireland, Galway. These modules involve students listening to stories (not taking traditional histories) from inpatients, reflecting on them and then presenting for discussion in a small-group environment.

There is a relative dearth of evidence for the impact of medical humanities teaching on future doctor performance. Ousager and Johannessen carried out a literature review covering 245 articles written between 2000 and 2008. ([ Ousager and Johannessen 2010)

The results showed that just 9 articles provided evidence of attempts to document the long–term impacts of teaching medical humanities modules to undergraduates. As the authors note, few aspects of medical education are able produce empirical evidence of their value in training doctors, and, in medical humanities perhaps more than other courses, it is challenging to identify measurable learning outcomes. However, not unreasonably, they warn that, in an era of evidence-based medicine, this lack of evidence could threaten the continued development of medical humanities courses in medical education.

Barber and Moreno-Leguizamon, in their paper “Can narrative medicine education contribute to the delivery of compassionate care? A review of the literature” ( Barber and Moreno-Leguizamon 2107) looked at whether there was sufficient evidence to demonstrate that narrative medicine education resulted in compassionate care. They concluded that

“Although the studies suggest that Narrative Medicine is beneficial, there is insufficient large-scale data to establish a higher clinical value. This is because there is a paucity of evidence demonstrating any behavioural outcomes in terms of follow-ups to individuals trained in narrative medicine or their long-term assessment, let alone the impact on patients.”

The findings in this review are therefore in keeping with previous literature reviews concerning results in humanities-based education: illustrating a beneficial effect on communication between doctors and patients, and personal growth including self-reflection and enjoyment in learning narrative medicine.

However it is time to approach this evidence deficit and do the “heavy lifting” required to show that narrative medicine, and by extension the medical humanities, make a difference to professional physician behaviour and patient outcome. The necessary research will require funding, time (long term follow-up) and determination.

As Blease suggests it is time for us to show instrumental value: “there are insidious consequences of placing the humanities on a lofty pedestal, where they can be admired but do no heavy lifting, where they are above the workmanship of application.” ( Blease 2016)

She speaks of the medical humanities “stick(ing) their head above the parapet.” This involves exposing ourselves to (at least) friendly fire. Which chimes with my call for more direct involvement of medical teachers and researchers if the humanities are to find an evidence- based place in medical education.

## Conclusion

Future research must, unashamedly, focus on being relevant for day- to- day practice. Humanists and doctors need to construct a narrative medicine/medical humanities curriculum that reflects the realpolitik of modern medical practice and the need for students to retain a “lean” set of skills suitable for practical use on busy wards and overbooked clinics.

Patients must be part of planning as well as executing this research. We work in an era when patienthood is the overarching goal of medical education. But above all, the time has come to promote medical humanities from being a soft topic to one that is “hardened” by incorporating it into the core curriculum.

Let’s join together in developing a “how to” exercise aimed at mainstreaming medical humanities.

## Take Home Messages

• Medical Humanities has a key role in the education of medical students
• It must be brought in from the cold of being an optional part of the curriculum optional
• Medical professionals need to take greater ownership of the discipline
• The lack of research into whether it changies medical practice in graduates must be addressed

## Notes On Contributors

Dr Muiris Houston is especially interested in the stories patients tell, which led him to complete a Masters in Medical Humanities at the University of Sydney, Australia. Muiris is adjunct assistant professor of medical humanities at Trinity College Dublin; he also teaches a narrative medicine module to medical students at NUI Galway. He is writing his first book at present. A graduate of Trinity College Dublin medical school, Dr Houston is on the Medical Council specialist register for both occupational medicine and family practice. He is also an award-winning medical journalist.
