# Supplementary material for: Medical Humanities: time to join the mainstream of medical education
Source: MedEdPublish (2016). 2018 Sep 3;7:182. [Version 1] doi: 10.15694/mep.2018.0000182.1 (PMC10701829; doi:10.15694/mep.2018.0000182.1)
Supplement: Peer review history

# Reviewer report: Reviewer response for version 1

**DOI:** 10.21956/mep.19661.r29339

**Published:** 2018-09-19

**Authors:** Jonathan McFarland

**Affiliations:** 1 Sechenov University, Moscow

This review has been migrated. The reviewer awarded 4 stars out of 5 I greatly appreciated this paper, finding it to be like a "wake up call" for all those interested in seeing the Humanities take their place at the core of medical education. So, I am in complete agreement with the sentiment of the paper, agreeing that now is the time to act and combat this creeping reductionism that is rife in medical education and practice, and as the author says so clearly we MUST not let Medical Humanities languish in the periphery, like a "Cinderella" discipline. It needs to be at the centre as it has a crucial role in "humanizing" future clinicians. I also applaud the author's focus on the patient role in both planning and executing the task ahead. I could not agree more. As the author mentions one of the main issues is the lack of evidence, which, along with the how, both needed to be seriously addressed in order to move forward. I would recommend that this article should be read by all interested in this topic. Thank you.

Reviewer Expertise:

NA

No decision status is available

# Reviewer report: Reviewer response for version 1

**DOI:** 10.21956/mep.19661.r29340

**Published:** 2018-09-14

**Authors:** Hedy S. Wald

**Affiliations:** 1 Warren Alpert Medical School of Brown University

This review has been migrated. The reviewer awarded 4 stars out of 5 Thank you Dr. Houston for delineating some key issues regarding implementation of medical humanities (MH) curriculum in health professions education. Within curriculum time constraints, what will be the convincing "evidence" for health professions education deans to find or create space to thread this into the curriculum rather than solely offering electives(s)? This is where the call for evidence of its value as you describe can be of help. The paper could be strengthened with some elaboration of this point, with literature showing benefits of including MH within health professions education. There has been some work done on narrative medicine and empathy for example, Chen et al 2016, BMC Med Educ). A hot off the press Commentary on MH in medical education and practice (Wald et al, 2018 Med Teacher) may have some helpful content. Consider the reference in that Commebtary to Peterkin's work on integrating MH within science curriculum... The paper may also be strengthened with some inclusion of the author's own experience and some concrete details on the Trinity College/National University of Ireland module that is mentioned. Of note, when citing the following, "Most clinicians are not scientists; they have a different responsibility – to attempt to relieve distress and suffering ....” (Heath, 2016), please consider the view that all clinicians should be scientists and approach their practice with rigor even while attempting to relieve distress and suffering...these can co-exist and should without an artificial divide. This may be generalized to the inclusion of MH as you have suggested-assessment can be part of the implementation strategy and is integral to curriculum development as per Kern's work. Check out Wald et al 2015 Acad Med for an example EHR curriculum that integrates narrative medicine! A tangible exemplar.There appears to be a missing reference for Wald, 2011-Appreciate the citation! We are on the cusp of some valuable MH inroads, thanks for contributing to this vital conversation!

Reviewer Expertise:

NA

No decision status is available

# Reviewer report: Reviewer response for version 1

**DOI:** 10.21956/mep.19661.r29347

**Published:** 2018-09-11

**Authors:** David Taylor

**Affiliations:** 1 Gulf Medical University, Ajman, UAE

This review has been migrated. The reviewer awarded 3 stars out of 5 This article is timely, but highlights for me a key issue. I think that the humanities can flourish in a curriculum where there are sufficient people involved in helping the students engage in them. There is a need for colleagues with formal training in the humanities, but in my experience, it is respected clinical colleagues who have the greatest impact through role modelling. It would be important to form, or more likely, strengthen the links between both sets of people to ensure there is a coherent approach and visible mutual respect.Patient narratives are one way forward, and I applaud the idea from Trinity College Dublin, but students need help to resolve the difference between then narrative and the clinical situation. This demands time which seems to be in increasingly short supply – or more precisely demands that the time given to helping junior colleagues come to an understanding of narrative is time well spent.

Reviewer Expertise:

NA

No decision status is available

# Reviewer report: Reviewer response for version 1

**DOI:** 10.21956/mep.19661.r29343

**Published:** 2018-09-05

**Authors:** Gert Van Zyl

**Affiliations:** 1 University of the Free State

This review has been migrated. The reviewer awarded 3 stars out of 5 A very important topic addressed and solid arguments made for it to become an important component in the curriculum of health sciences. Health science educators and those involve din curriculum development and design will benefit to learn from the concepts in the article. Can make a future impact on where "we are at present" and where "we need to go".

Reviewer Expertise:

NA

No decision status is available

# Reviewer report: Reviewer response for version 1

**DOI:** 10.21956/mep.19661.r29342

**Published:** 2018-09-04

**Authors:** Julie Browne

**Affiliations:** 1 Cardiff University School of Medicine

This review has been migrated. The reviewer awarded 3 stars out of 5 I couldn’t agree more with the aims of this article; medicine is an art as much as a science and there is increasing evidence to show that medical students, and the patients for whom they will care in future, could benefit from “a greater involvement in, and commitment to, the medical humanities by mainstream medical educators.” The example given of how patient narrative is currently treated and what might change if a more humanities approach to narrative were adopted is a fine example of the way in which medical education could benefit from an approach that is driven by some of the basic concepts in humanities.However, I am a little cautious about the conclusions drawn by the paper – that it is time to show that “narrative medicine and … medical humanities make a difference to professional physician behaviour and patient outcome”. These are fine aims, but by proposing these arguments we may paradoxically be weakening our justification for including art in the mainstream medical curriculum. Art will never have the objectively measurable influence on health that those who oppose its inclusion would require. I agree with Bleakley (2017) that the argument that the purpose of medical humanities should be to improve health is, in fact, a fallacy in any case. Bleakley reminds us that the point of art has always been to disrupt; to “ruffle feathers and upset preconceptions”. Moreover, as he observes, art is sometimes therapeutic - but can sometimes drive people to fury, frustration and bafflement. And it’s not always narrative based. Art is so much more than any of these utilitarian arguments would suggest. And many of the key arguments we use to defend the place of humanities may actually be limiting their importance as a fundamental human need.Let us therefore be cautious in our discussions around the place of art and humanities in the medical curriculum. We need to ensure that we are not selling our students and their patients (not to mention art itself) short, by attempting to make a workhorse out of a unicorn. https://onlinelibrary.wiley.com/doi/abs/10.1111/medu.13236

Reviewer Expertise:

NA

No decision status is available

# Reviewer report: Reviewer response for version 1

**DOI:** 10.21956/mep.19661.r29345

**Published:** 2018-09-04

**Authors:** P Ravi Shankar

**Affiliations:** 1 American International Medical University

This review has been migrated. The reviewer awarded 4 stars out of 5 Thank you for the opportunity to read and review this interesting paper. I agree with the author that Medical Humanities (MH) is most often at the fringes of medical education. When I first introduced a MH module at a Nepalese medical school it was as a voluntary module. Future modules were mandatory and facilitated by clinical educators and psychologists but were not really at the center of the curriculum. MH is often regarded as something which is good to know, appreciated by accreditors but not really at the center of things. I agree with the author that providing evidence for the effectiveness of MH in the short, medium and long term is vital. Curricular decisions are often based on evidence of effectiveness and it is time that evidence for the effectiveness of MH is produced. There are a number of challenges in this regard as has been pointed out by the author. There are a few spelling errors which could be corrected. This paper will be of interest to all curriculum developers and medical educators and of special interest to those facilitating MH modules.

Reviewer Expertise:

NA

No decision status is available

# Reviewer report: Reviewer response for version 1

**DOI:** 10.21956/mep.19661.r29341

**Published:** 2018-09-03

**Authors:** Benjamin Burrows

**Affiliations:** 1 NHS

This review has been migrated. The reviewer awarded 3 stars out of 5 An enjoyable and timely article that highlights a long standing issue with medical humanities. Whilst it is seen as a soft skill or even an ancillary skill, its role in medical education will fail to be utilised. As the author correctly states its subject matter seems to be squeezed between the traditional subjects such as physiology, pharmacology, pathology and so on.As someone involved with teaching medical humanities in my local University, I have found my combination of clinical experience with the knowledge of humanities has really interested the students and focussed their attention to its importance.Until the subject receives its rightful place future clinicians will be doomed to make the same mistakes as those who trod before us. How we do this though, still seems elusive.

Reviewer Expertise:

NA

No decision status is available

# Reviewer report: Reviewer response for version 1

**DOI:** 10.21956/mep.19661.r29344

**Published:** 2018-09-03

**Authors:** Nandalal Gunaratne

**Affiliations:** 1 Faculty of Medicine, Wayamba University of Sri Lanka

This review has been migrated. The reviewer awarded 4 stars out of 5 This is a good article and timely as medical Humanities are increasingly being discussed and promoted in medical curricula. However the authors have not given suggestions of how it can be integrated into the mainstream curriculum.Sometimes cthe impact of ertain things are difficult to measure. Like love. Heisenbergs uncertainty principle showed we cannot measure something without disturbing it, but the disturbance and measurement were both real.So it is with humanities. It's need is real. It's impact difficult to fathom.

Reviewer Expertise:

NA

No decision status is available

# Reviewer report: Reviewer response for version 1

**DOI:** 10.21956/mep.19661.r29346

**Published:** 2018-09-03

**Authors:** Trevor Gibbs

**Affiliations:** 1 AMEE

This review has been migrated. The reviewer awarded 3 stars out of 5 An interesting paper that adds to our call for more Humanities within the undergraduate medical and I feel healthcare curricula. I think many who advocate such change would agree wholeheartedly with the author's views. I specifically appreciate the call for a more "so what" element to the introductions of the Humanities- we have to show / prove that adding the Humanities produces "better" doctors, equipped to provide a healthcare service appropriate for the changing needs of the population, however big, however diverse and wherever placed globally. I still feel that there is still more to be done in deciding on the appropriate way to integrate the Humanities into our curricula, so I feel that this paper could have been improved somewhat by including a brief description of how the author included it into the Trinity course- and expanding on how we evaluate such actions, perhaps these are the subject of other papers; we need to be practical as well as enthusiastic in our approaches.

Reviewer Expertise:

NA

No decision status is available
